# Supplementary material for: A redox-based electrogenetic CRISPR system to connect with and control biological information networks
Source: Nat Commun. 2020 May 15;11:2427. doi: 10.1038/s41467-020-16249-x (PMC7228920; doi:10.1038/s41467-020-16249-x)
Supplement: Supplementary file 1 — Supplementary Information [file 41467_2020_16249_MOESM1_ESM.pdf]

A redox-based electrogenetic CRISPR system to connect with and control biological information networks

Bhokisham et al.

Supplementary Information

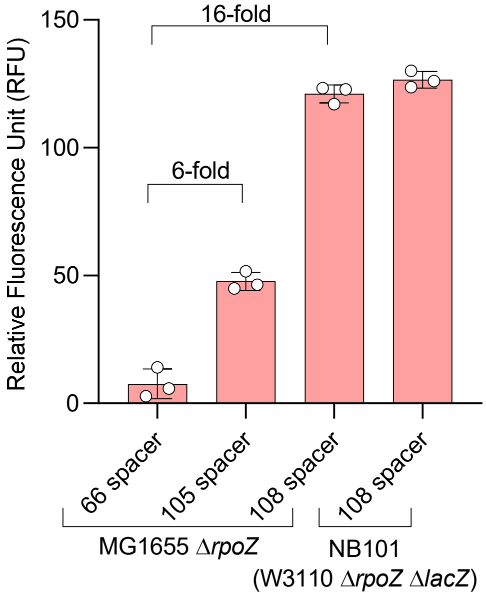

Supplementary Figure 1: Comparison of CRISPR activation obtained from NB101 strain (*E. coli* W3110 *lacZ*$^{-}$ *rpoZ*$^{-}$) and MG1655 *rpoZ*$^{-}$ strain

CRISPRa experiment is performed by transforming plasmids expressing spacers (105, 108 and 66 spacer) and target plasmid (pWJ89) into *E. coli* MG1655 *rpoZ*$^{-}$ strain, as detailed in Bikard *et. al*. 108 spacer is also tested in the newly created NB101 (*E. coli* W3110 *lacZ*$^{-}$ *rpoZ*$^{-}$) strain and CRISPRa is measured via GFP transcriptional activation from pWJ89 using a plate reader 16 hours after re-inoculation. Independent experimental replicates (n=3) are represented by circles, height of the bars indicate mean and error bars indicate standard deviation. Source data is provided separately.

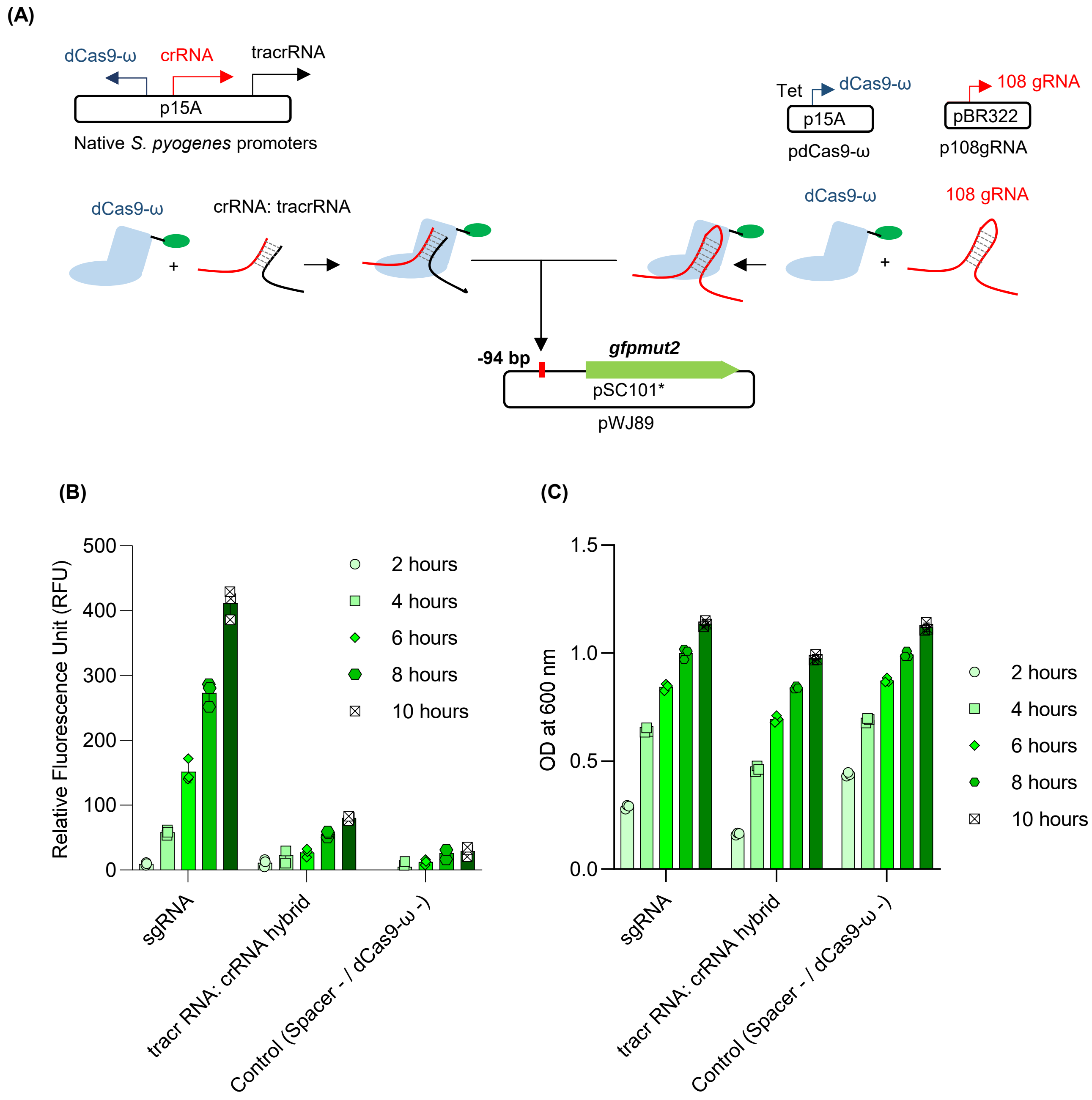

Supplementary Figure 2: Comparison of CRISPR activation from tracrRNA:crRNA and gRNA based delivery systems

Scheme of the CRISPR-Cas9 mediated transcriptional activation (CRISPRa) of GFP in target plasmid pWJ89 using tracrRNA:crRNA and gRNA systems for spacer delivery **(A)**. With the tracrRNA:crRNA hybrid system, spacers and dCas9-ω are expressed from native *S. pyogenes* promoters. In the gRNA system, spacers are expressed as short gRNA under constitutive J23119 promoters from p108gRNA and dCas9-ω from the Tet promoter in pdCas9-ω under leaky expression conditions. **(B)** Fluorescence and **(C)** OD_{600} is measured using a plate reader across 10 hours after re-inoculation. In all figs, independent experimental replicates (n=3) are represented by corresponding symbols as indicated in the legend, height of the bars indicate mean and error bars indicate standard deviation. Source data is provided separately.

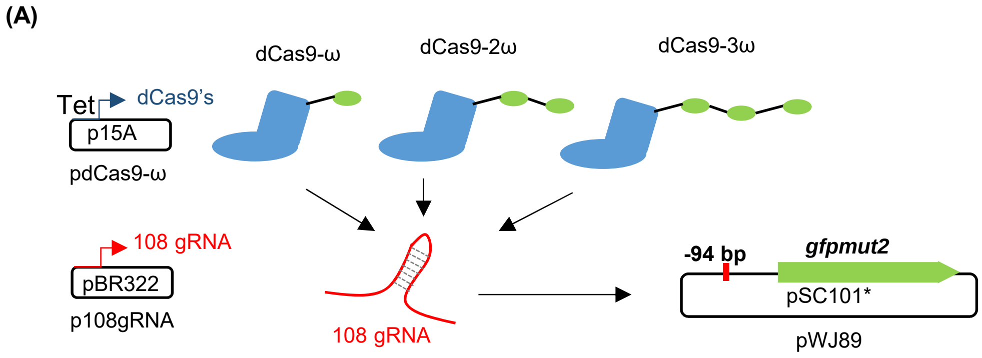

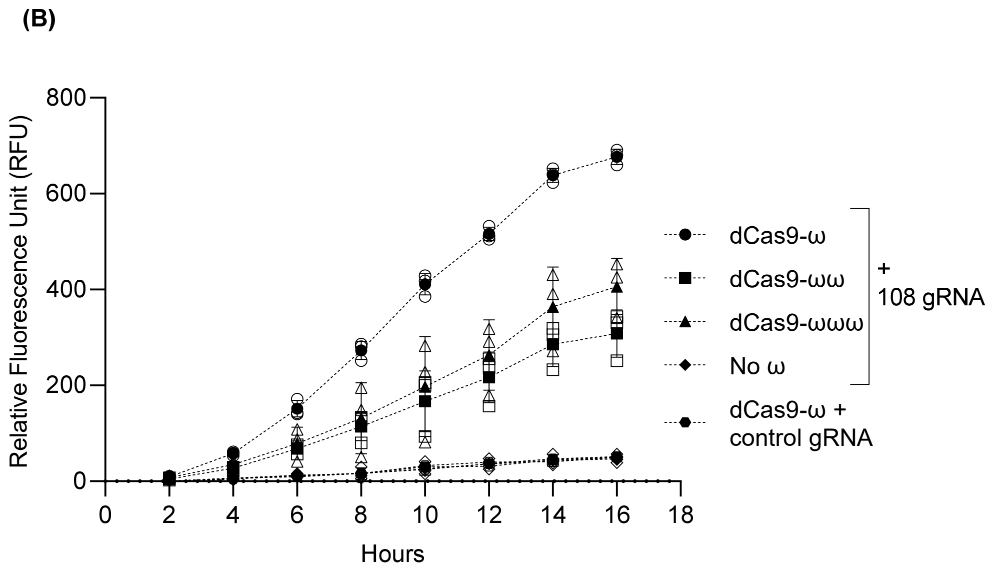

Supplementary Figure 3: Role of the number of ω subunits fused to dCas9 on CRISPRa in bacteria

(A) Comparison of the effect of the number of ω subunits fused to the C termini of *S. pyogenes* dCas9 for CRISPRa. dCas9-ω, dCas9-2ω and dCas9-3ω's are placed under a Tet promoter and used under leaky expression conditions. All dCas9's are directed towards GFP in pWJ89 using 108 gRNA for CRISPRa in NB101 cells. **(B)** GFP fluorescence is measured using a plate reader indicating transcriptional activation over 16 hours post re-inoculation. Independent experimental replicates (n=3) are represented by empty symbols as indicated in legends. The mean value is represented by the corresponding filled symbols and error bars indicate standard deviation. Source data is provided separately.

Introduction of mismatches in gRNA for a tunable CRISPRa system

Here, we examined whether mismatches in the gRNA seed region might play a role in tuning CRISPRa. Single point mutations in the seed region of a spacer sequence have been shown to significantly reduce the efficiency of CRISPRi.[1] To test if this factor plays a significant role in CRISPRa as well, we introduced single point mutations by replacing purines with pyrimidines or vice versa in the seed region to create non-cognate gRNAs NC-1, 4 and 5 expressed under J23119 promoter in pNC-gRNA 1, 4 and 5 respectively. We transformed the non-cognate gRNA plasmids along with pdCas9-ω and pWJ89 plasmids into NB101 cells and measured the effects of non-cognate gRNA on GFP activation. We measured GFP fluorescence levels at 4, 8 and 24 hours after re-inoculation and results indicated that under the conditions tested, non-cognate gRNAs had no significant effect on CRISPRa of GFP at 4 and 8 hours (Supplementary Figure 4).

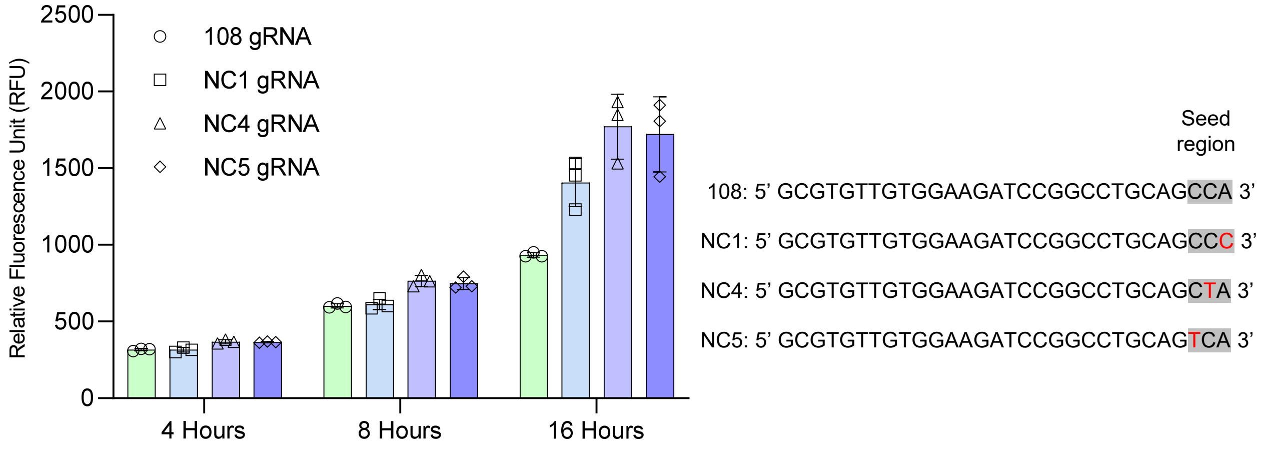

Supplementary Figure 4: Effect of point mutations in the seed region of gRNA on CRISPRa in bacteria
3 non-cognate gRNAs (denoted as NC1, NC4 and NC5) with single point mutations (denoted in red) in the seed region of the cognate 108-spacer sequence are expressed under J23119 promoters in pNC-gRNA 1, 4 and 5 respectively to target GFP (pWJ89) for CRISPRa in NB101 cells. dCas9-ω is used under leaky expression conditions from the Tet promoter for CRISPRa. GFP fluorescence is measured via a plate reader at 4, 8 and 24 hours post re-inoculation. Independent experimental replicates (n=3) are represented by corresponding symbols as indicated in legends. Height of the bars indicate mean and error bars indicate standard deviation. Source data is provided separately.

Regulating dCas9 expression from Tet promoters for a controllable CRISPRa system

To create a controllable CRISPRa system, we intended to regulate expression of dCas9-ω under the Tet promoter in pdCas9-ω. To the NB101 cells containing p108gRNA, pdCas9-ω and pWJ89 plasmids, we added varying concentrations of inducer, aTc, from 100 nM to 10 pM at the time of reinoculation and grew at 37°C. After 6 hours, we measured the relative gene expression levels of dCas9-ω and GFP through qPCR (dCas9-ω levels were determined by *rpoZ* coding for ω subunit of the dCas9-ω fusion in a *rpoZ*$^{-}$ strain). In addition to the relative gene expression levels of dCas9-ω and GFP (Supplementary Figure 5A), we measured GFP fluorescence (Supplementary Figure 5B) and cell growth (OD_{600}) (Supplementary Figure 5C) at 4 and 8 hours.

qPCR data indicated that only the 100nM aTc concentration condition significantly increased the dCas9-ω expression levels (indicated by *rpoZ* mRNA) over leaky expression from the Tet promoter. However, the *gfpmut2* expression data at 6 hours showed a markedly different gene expression pattern with the 100 pM condition displaying the highest *gfpmut2* expression. aTc concentrations higher than 100 pM had lower *gfpmut2* levels. GFP fluorescence and OD_{600} (Supplementary Figure 5B and 5C) showed a consistent trend of decreasing GFP fluorescence with increasing concentrations of aTc at both 4- and 8-hours post inoculation. While there was no significant increase in dCas9-ω expression with increasing aTc concentration (from 0 M to 10 nM), there was still a consistent decrease in GFP fluorescence and OD_{600} suggesting toxicity due to dCas9 induction.

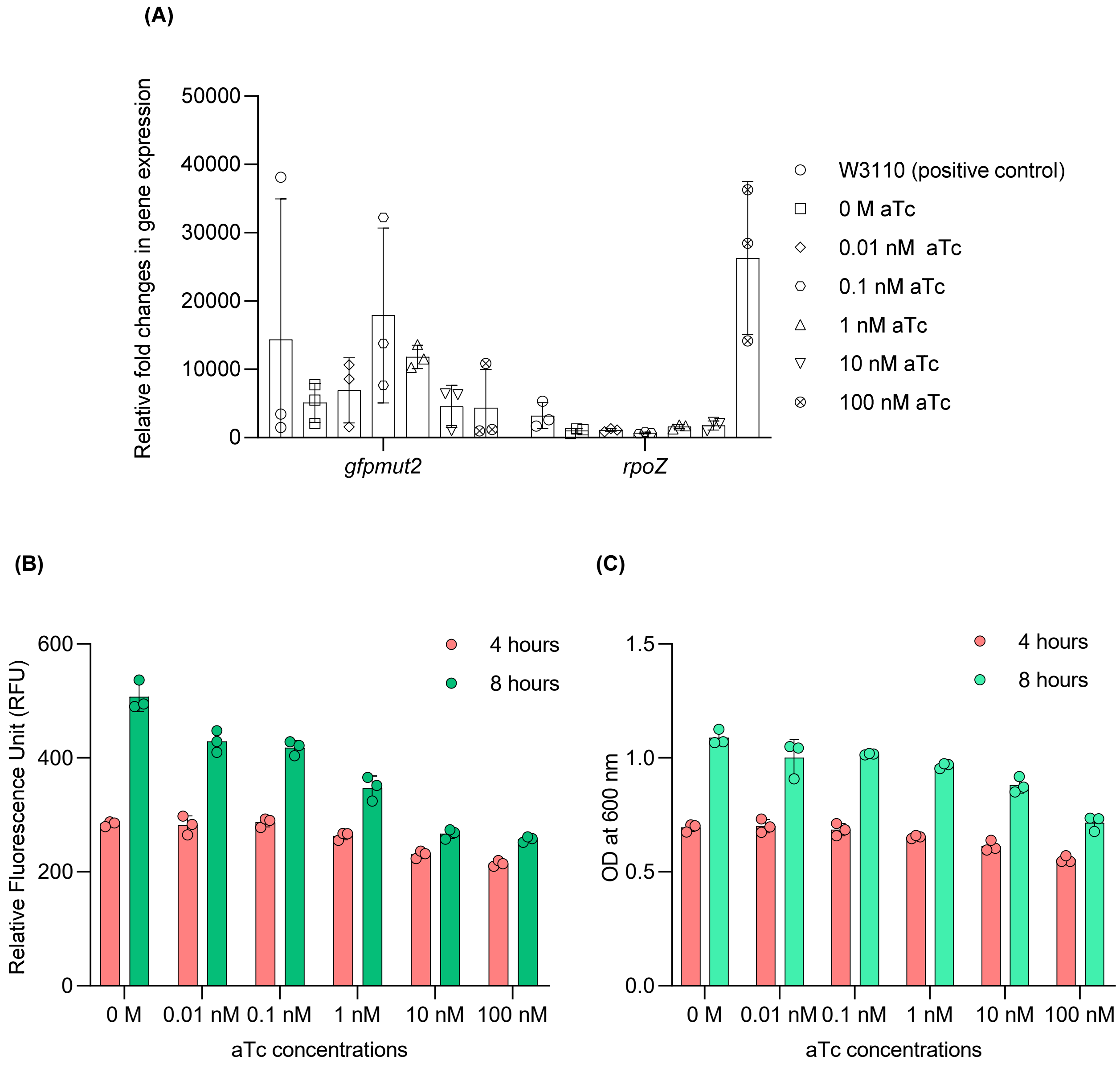

Supplementary Figure 5: Regulating dCas9 expression from Tet promoters for a controllable CRISPRa system

Plasmids pdCas9-ω, p108gRNA and pWJ89 are transformed into NB101 cells and the Tet promoter in pdCas9-ω is induced with different aTc concentrations during reinoculation for dCas9-ω expression. **(A)** qPCR data indicating relative expression levels of *dCas9-ω* and *gfp* measured 6 hours after addition of aTc and normalized to the negative control. *rpoZ* corresponds to the ω subunit and indicates relative levels of dCas9-ω fusions. **(B)** and **(C)** indicates GFP fluorescence and OD_{600} data obtained 4 and 8 hours after addition of various concentrations of aTc to induce dCas9-ω expression. Independent experimental replicates (n=3) are represented by corresponding symbols as indicated in legends. Height of the bars indicate mean and error bars indicate standard deviation. Source data is provided separately.

Engineering of promoter strength and ribosomal binding site for a controllable dCas9 expression from Tet promoters:

To make the dCas9-ω based CRISPRa system a controllable and inducible system wherein CRISPRa of target genes occurs only after induction of dCas9-ω, we attempted to regulate expression of dCas9-ω by engineering both its transcription and translation. To attenuate accumulation from leaky expression of dCas9-ω from the Tet promoters, we added a protein degradative ssrA tag to the C terminus of the dCas9-ω fusion (pdCas9-ω_{ssrA}, Supplementary Figure 6B). In addition, we removed the natural promoters for the Tet Repressor and replaced them with a stronger synthetic promoter for Tet repressor to increase the expression of the Tet repressor and, in turn, increase repression of the Tet promoter. In addition, we reduced ribosome binding site (RBS) strength by ~10 fold for dCas9-ω from the Tet promoter. These measures were previously used to reduce leaky expression of Cas9 resulting in increased genome editing efficiencies.[3] Promoter and RBS changes were introduced into both pdCas9-ω and pdCas9-ω_{ssrA} to create pIntdCas9-ω (Supplementary Figure 6C) and pIntdCas9-ω_{ssrA} (Supplementary Figure 6D). We expected these changes would make dCas9-ω mediated CRISPRa more inducible.

We transformed these dCas9 expression plasmids with p108gRNA and pWJ89 plasmids in NB101 cells and induced with varying concentrations of aTc at the time of inoculation and measured GFP fluorescence at 6 hours. Under the uninduced conditions (i.e., no aTc), CRISPRa of GFP decreased with increasing constraints on the transcription and translation of dCas9-ω. CRISPRa from pdCas9-ω was the highest followed by the activation from pdCas9-$\omega_{ssrA.}$ CRISPRa levels from pIntdCas9-ω and pIntdCas9-ω_{ssrA} were even lower (Supplementary Figure 6E). These results indicated that the changes introduced both at protein and translational levels (addition of ssrA tags to dCas9-ω and reduced RBS strength for dCas9-ω, respectively) as well as transcriptional level (increased promoter strength for the Tet

Repressor) reduced the leaky expression of dCas9-ω. Yet, at the same time, our efforts to make the Tet promoter controllable where upon addition of aTc there should be an increase in CRISPRa of GFP, were unsuccessful. There was no increase in transcriptional activation of GFP with addition of aTc in any of the conditions except in the case of pdCas9-ω_{ssrA} where 1nM aTc resulted in a minor increase. These results suggested that while efforts to contain leaky expression of dCas9-ω was successful, our efforts were unsuccessful for developing controllable dCas9-ω mediated activation of GFP. In subsequent experiments, we continued to use the leaky Tet promoter in pdCas9-ω.

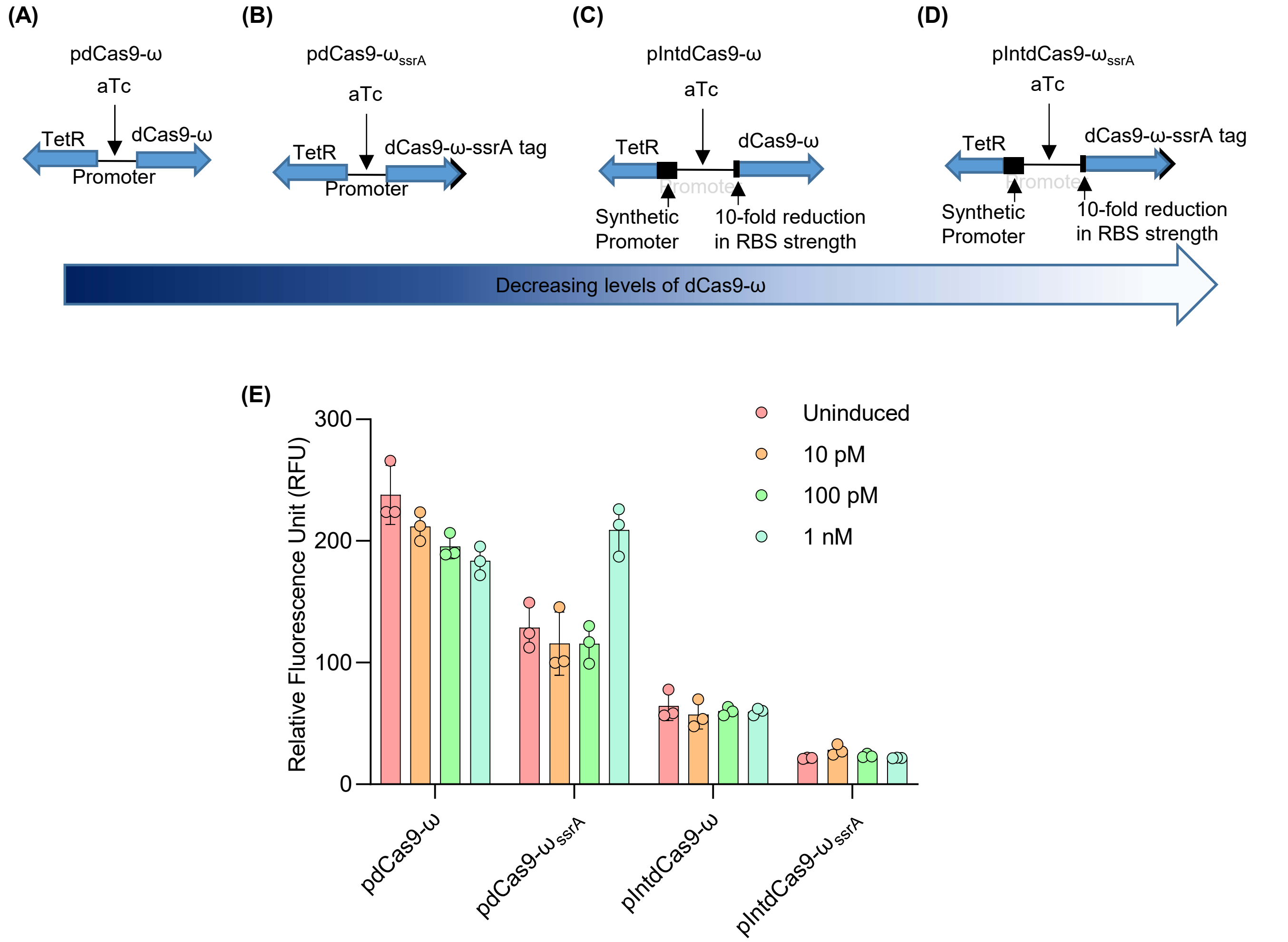

Supplementary Figure 6: Engineering dCas9-ω transcription and translation processes to limit leakiness and control expression

(A) Scheme indicates Tet promoter with dCas9-ω in pdCas9-ω. **(B)** In addition to (A), the dCas9-ω is engineered with a degradative ssrA tag at the C termini of dCas9 (pdCas9-ω_{ssrA}). **(C)** Scheme of engineered synthetic promoter for TetR to upregulate expression of Tet repressor and an engineered RBS site with 10-fold reduction in RBS site strength (pIntdCas9-ω). **(D)** In addition to (C), a ssrA tag is engineered to the C termini of dCas9-ω (pIntdCas9-ω_{ssrA}). **(E)** All the different dCas9-ω plasmids are transformed into NB101 cells along with pWJ89 and p108gRNA and the resulting GFP fluorescence is measured via a plate reader 6 hours after reinoculation. Different aTc concentrations are added during reinoculation as well. Independent experimental replicates (n=3) are represented by circles as indicated in legends. Height of the bars indicate mean and error bars indicate standard deviation. Source data is provided separately.

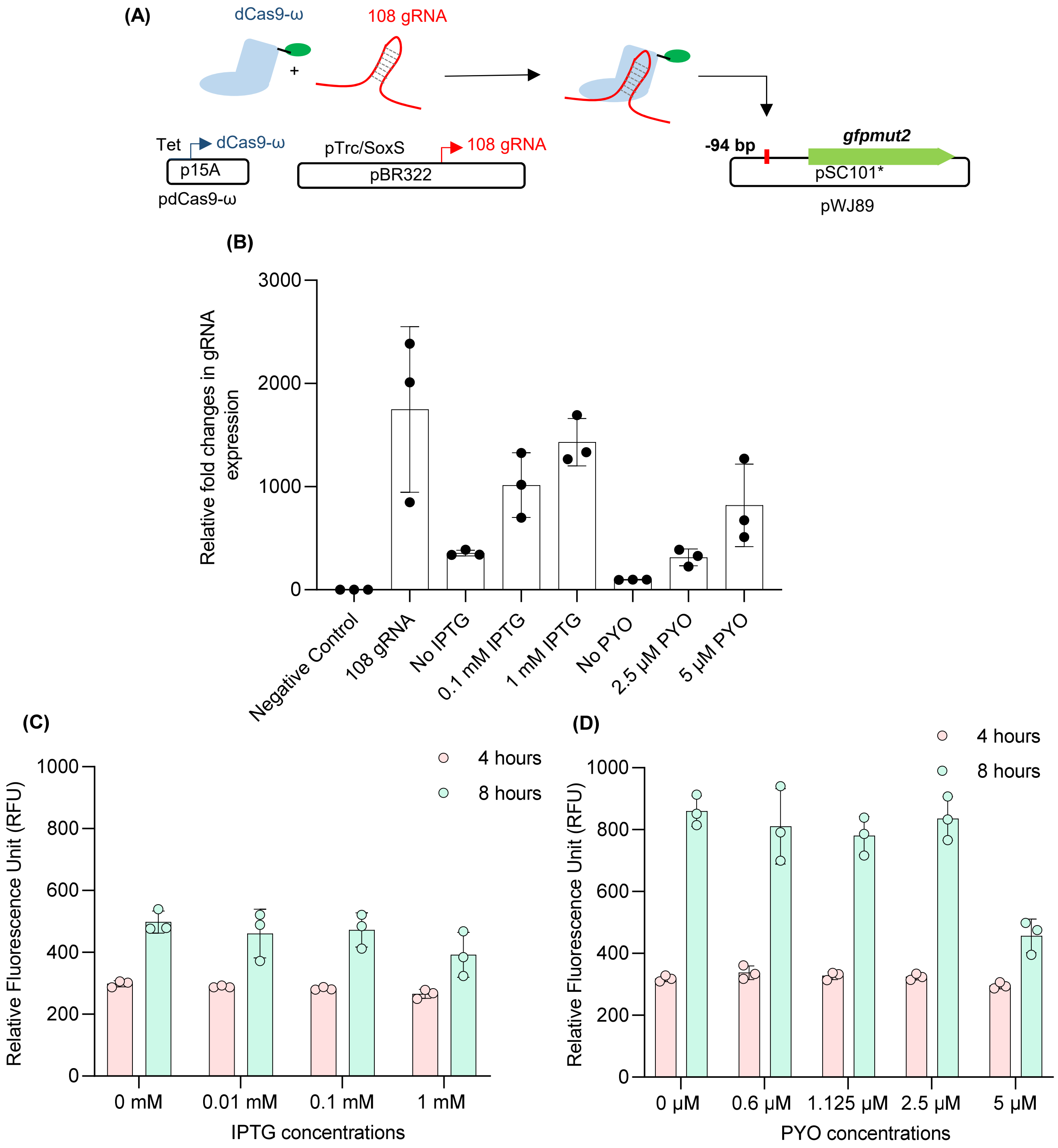

Supplementary Figure 7: Controlling gRNA expression from inducible promoters for a tunable and inducible CRISPRa response

(A) Scheme of controlling gRNA expression from the inducible pTrc and pSoxS promoters to mediate a tunable and inducible CRISPRa response. pTrc-108gRNA and pSoxS-108gRNA promoters are transformed along with pWJ89 and pdCas9-ω into NB101 cells. (**B)** pTrc and pSoxS promoters are induced with varying concentrations of inducers (IPTG and Pyocyanin for pTrc and pSoxS respectively) and qPCR is performed after 6 hours to measure relative gRNA expression levels. Negative control is mRNA from an *E. coli* strain lacking gRNA expression and positive control is gRNAs expressed under J23119 constitutive promoter from p108gRNA in NB101 cells. CRISPRa resulting from the gRNA expression induced from pTrc **(C)** and pSoxS **(D)** promoters is measured by GFP fluorescence at 4 and 8 hours post gRNA induction. Independent experimental replicates (n=3) are represented by circles as indicated in legends. Height of the bars indicate mean and error bars indicate standard deviation. Source data is provided separately.

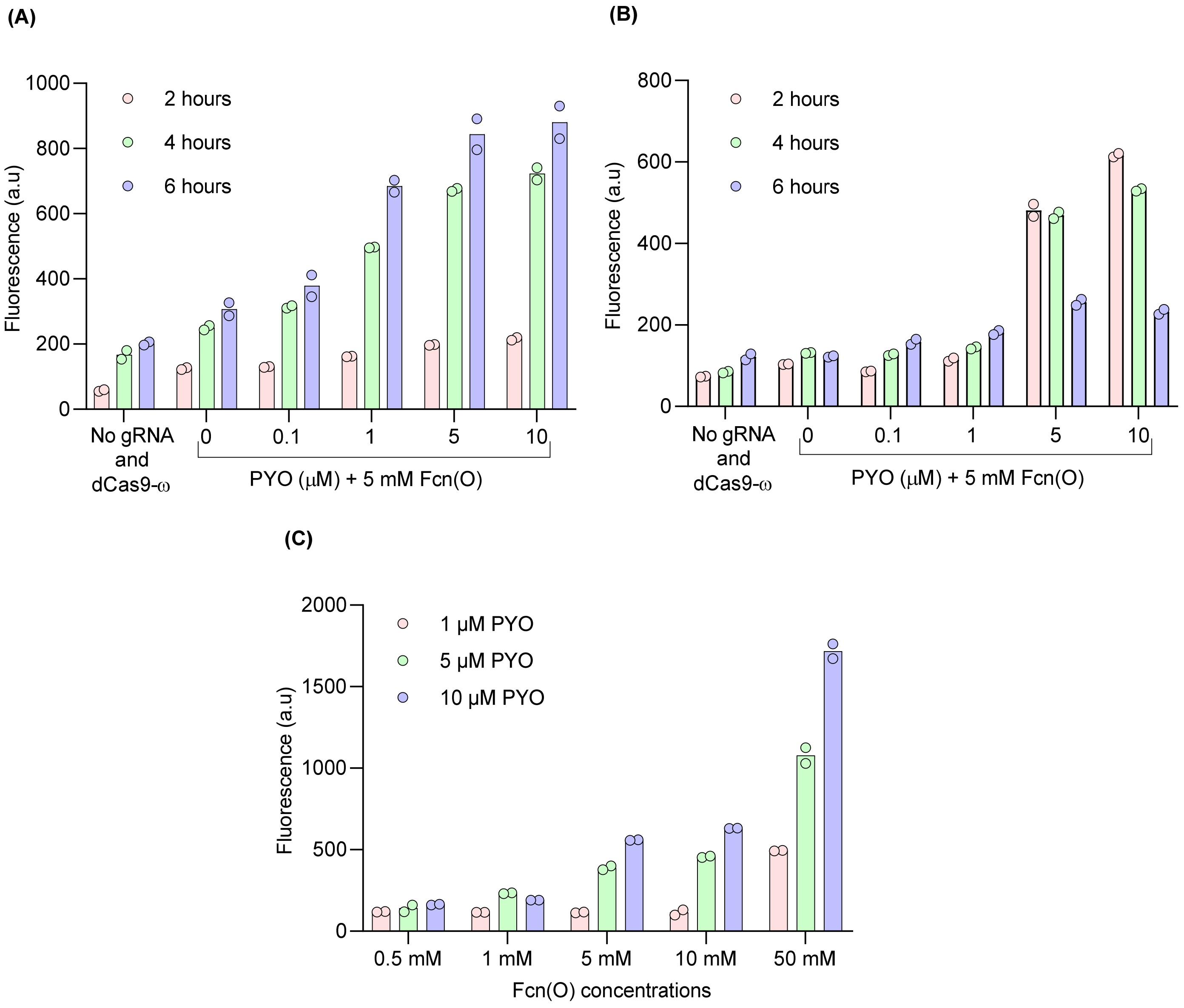

Supplementary Figure 8: Ferricyanide and Pyocyanin mediated control of SoxS promoters for gRNA expression and CRISPRa

NB101 cells are transformed with pSC-S108gRNA, pMC-phiLOV and pdCas9-ω and grown in LB media to OD_{600} 0.6. Later, cells are re-suspended in minimal-M9 media with various concentrations of Fcn(O) and PYO and grown under aerobic or anaerobic conditions at 37ºC. With a constant Fcn(O) concentration of 5 mM, PYO concentrations are varied and the resulting CRIPSRa with cells in both aerobic **(A)** and anerobic **(B)** conditions is measured via phiLOV fluorescence. **(C)** PYO and Fcn(O) concentrations are simultaneously varied and the resulting CRISPRa is measured via phiLOV fluorescence in anerobic conditions after 2 hours. phiLOV fluorescence is measured via flow cytometer. Independent experimental replicates (n=2) are represented by circles and height of the bars indicate mean. Source data is provided separately.

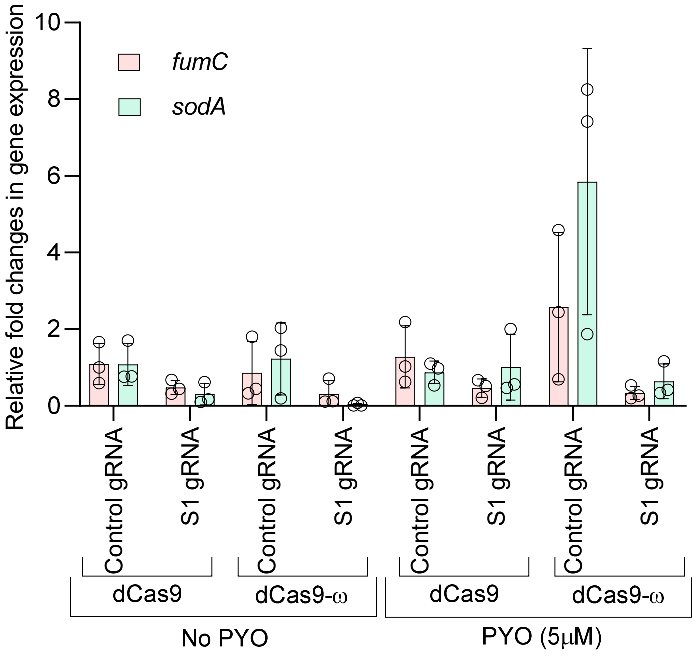

Supplementary Figure 9: CRISPR mediated repression of SoxS leading to indirect repression of SoxS derived oxidative stress defense responses.

PAM site in the *soxS* coding region (+4 in the non-coding strand) is targeted with S1 gRNA expressed under J23119 promoters from pS1gRNA. pS1gRNA is transformed into NB101 cells containing dCas9-ω and grown in LB media under aerobic conditions to OD_{600} 0.6. Later, 5 μM of PYO is added and after 3 hours of *soxS* induction, indirect repression of *sodA* and *fumC* is measured via qPCR. Independent experimental replicates (n=3) are represented by circles. Height of the bars indicate mean and error bars indicate standard deviation. Source data is provided separately.

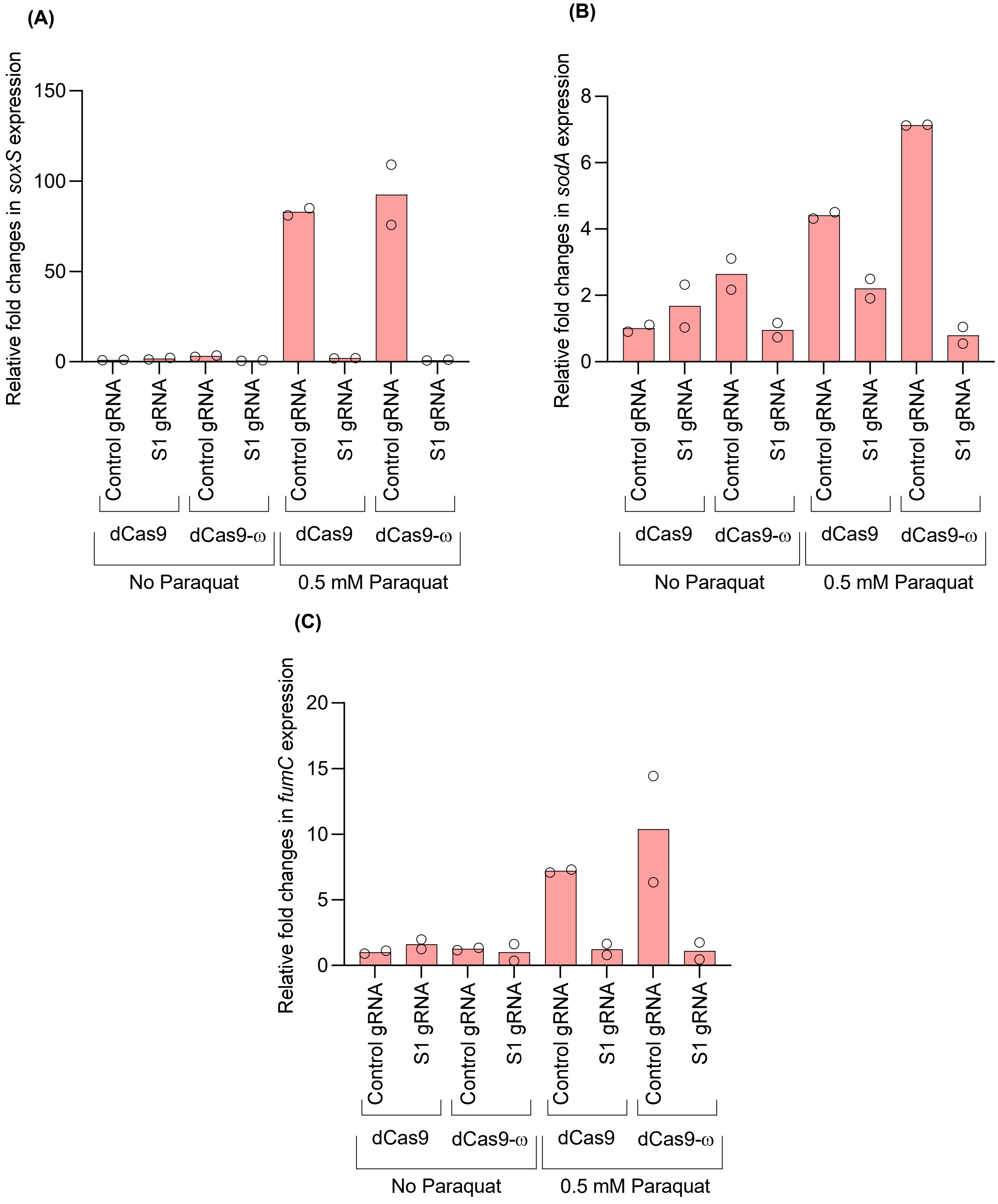

Supplementary Figure 10: Repression of SoxS and SoxS mediated oxidative stress response with Paraquat

(A), (B) and **(C)** indicate relative expression levels of s*oxS*, *sodA* and *fumC* respectively when repressed with S1 as well as Control gRNA. Both dCas9 as well as dCas9-ω is used for repression. *soxS* in *E. coli* genome is induced with 0.5 mM Paraquat and gene expression levels are measured via qPCR after 3 hours. Independent experimental replicates (n=2) are represented by circles and height of the bars indicate mean. Source data is provided separately.

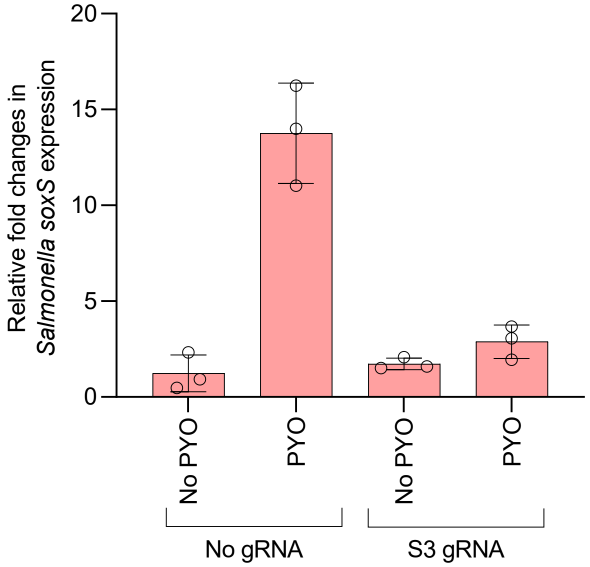

Supplementary Figure 11: CRISPR transcriptional activator mediated repression of *soxS* in *S. enterica*

A single PAM site in the *soxS* coding region (+29 bp in the non-coding strand of *soxS*) in *S. enterica* is targeted with S3 gRNA expressed under the J23119 promoter from pSoxS-phiLOV:pS3gRNA. Both pSoxS-phiLOV:pS3gRNA and pdCas9-ω are transformed into *S. enterica,* grown to OD_{600} 0.6 and induced with 5 μM of PYO under aerobic conditions. After 3 hours of induction, repression of *soxS* is measured via qPCR. Independent experimental replicates (n=3) are represented by circles. Height of the bars indicate mean and error bars indicate standard deviation. Source data is provided separately.

Ribozyme mediated simultaneous repression and activation:

Having shown that we could repurpose the dCas9 based transcriptional activator to repress genes, we sought to study whether the transcriptional activator could simultaneously perform CRISPRi and CRISPRa at different sites. With repression of *soxS* in the genome leading to a ~3-4-fold increase in SoxS promoter activity, we explored whether this enhancement in SoxS promoter activity could lead to an increase in gRNA expression and, in turn, CRISPRa. To demonstrate, we intended to express two gRNAs from the SoxS promoter to mediate CRISPRa of reporter genes as well as CRISPRi of *soxS* in the *E. coli* genome. For this purpose, we engineered both S1 gRNA targeting *soxS* in the genome and 108 gRNA targeting phiLOV in pMC-phiLOV into a single transcript and expressed it from the SoxS promoter in a plasmid denoted pSCSoxS-S1-108gRNA. To mediate gRNA processing, we introduced self-cleaving ribozyme hammerhead (HH)[4] at the 5' end of each gRNA and ribozyme hepatitis delta virus (HDV)[5,6] at the 3' end of each gRNA. We also built a construct (pSCSoxS-Control-108gRNA) expressing Control gRNA and 108 gRNA for controls (Supplementary Figure 12A). As additional controls for ribozyme processing, we also included pSC-S108gRNA plasmid expressing just the 108 gRNA for phiLOV activation.

We individually transformed these plasmids along with pdCas9-ω and target plasmid pMC-phiLOV into NB101 cells and grew the cells in LB media at 37^{0}C. At OD_{600} 0.6, we suspended the cells in minimal-M9 media and induced the cells with various concentrations of Fcn(O) and PYO under anaerobic conditions. We measured phiLOV fluorescence after 2 hours. Results indicated that there was phiLOV fluorescence in all conditions indicating that the 108 gRNA responsible for activation of phiLOV was functional (Supplementary Figure 12B). However, activation of phiLOV with pSCSoxS-S1-108gRNA (expressing S1 gRNA as well as 108 gRNA) was not higher than activation of phiLOV with 108 gRNA only (from pSC-

S108gRNA) indicating that there was no enhancement in CRISPRa arising from S1 gRNA mediated repression of *soxS* in the genome.

To analyze whether the S1 gRNA was functional post the ribozyme processing of S1-108 gRNA hybrids and repressed *soxS* in the genome as intended, we transformed pSCSoxS-S1-108gRNA plasmid along with pdCas9-ω into NB101 cells. Upon inoculation, we induced *soxS* by the addition of 5 μM PYO and measured *soxS* repression. As controls, we also had p108gRNA expressing 108 gRNA as well as pSCSoxS-Control-108gRNA. As expected, strains expressing *soxS* specific S1 gRNA alone displayed non activation of *soxS* in the genome upon PYO induction (Supplementary Figure 12C), indicating that the S1 gRNA expressed as a hybrid along with 108gRNA from the pSCSoxS-S1-108gRNA was functional. We then tested the effect of S1-108 gRNA hybrid on SoxS promoter activity. Results from Figure 4C indicated that the S1 gRNA, when expressed independently improved the activity of the SoxS promoter by ~3-4 fold. We expected the same from S1-108 gRNA hybrid as well. We transformed pSCSoxS-S1-108gRNA with pSoxS-phiLOV into NB101 cells. As controls, we also used pS1gRNA in combination with pSoxS-phiLOV and pdCas9-ω. We grew the cells as before and induced the populations with 5 mM Fcn(O) and 5 μM PYO for 2 hours under anaerobic conditions. Results showed that the pSCSoxS-S1-108gRNA plasmids did enhance the SoxS promoter activity by two-fold (Supplementary Figure 12D). These results indicated that S1 gRNA expressed in combination with 108 gRNA from pSCSoxS-S1-108gRNA plasmid was functional post the ribozyme processing. However, S1 gRNA when expressed in combination with another gRNA in multiplexed gRNA format gave just a 2-fold increase in SoxS promoter activity in comparison to 3-fold increase when expressed independently. In sum, we have demonstrated simultaneous CRISPR based repression and activation of two genes using a transcriptional activator dCas9-ω. We have also shown that while CRISPR based repression of *soxS* in the genome leads to 4-fold increase in SoxS promoter activity, similar increases in

CRISPRa was not achieved indicating the limitations of connecting CRISPRi of *soxS* with CRISPRa.

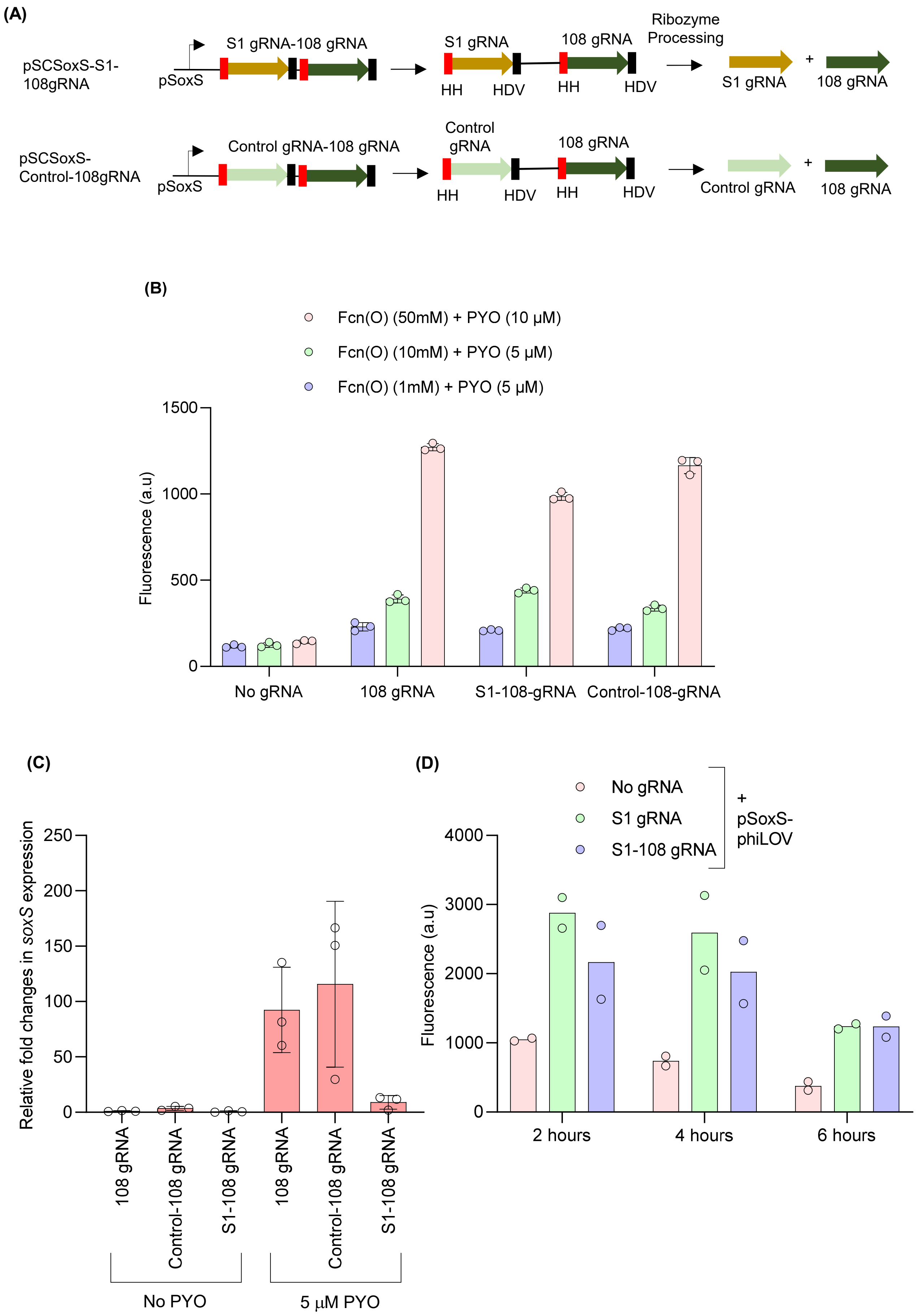
(A)
pSCSoxS-S1-108gRNA
pSoxS
S1 gRNA-108 gRNA
S1 gRNA
108 gRNA
HH
HDV
HH
HDV
Ribozyme Processing
S1 gRNA
+
108 gRNA
pSCSoxS-Control-108gRNA
pSoxS
Control gRNA-108 gRNA
Control gRNA
108 gRNA
HH
HDV
HH
HDV
Control gRNA
+
108 gRNA
(B)
Fcn(O) (50mM) + PYO (10 μM)
Fcn(O) (10mM) + PYO (5 μM)
Fcn(O) (1mM) + PYO (5 μM)
Fluorescence (a.u)
1500
1000
500
0
No gRNA
108 gRNA
S1-108-gRNA
Control-108-gRNA
(C)
Relative fold changes in soxS expression
250
200
150
100
50
0
108 gRNA
Control-108 gRNA
S1-108 gRNA
108 gRNA
Control-108 gRNA
S1-108 gRNA
No PYO
5 μM PYO
(D)
No gRNA
S1 gRNA
S1-108 gRNA
+ pSoxS-phiLOV
Fluorescence (a.u)
4000
3000
2000
1000
0
2 hours
4 hours
6 hours

Supplementary Figure 12: Simultaneous activation and repression of genes using CRISPRa

(A) Scheme of plasmids pSCSoxS-S1-108gRNA and pSCSoxS-Control-108gRNA. gRNA sequences are flanked with ribozymes hammerhead (HH) at the 5' end and hepatitis delta virus (HDV) at the 3' end to facilitate RNA processing. **(B)** Both plasmids are transformed into NB101 cells along with pdCas9-ω and pMC-phiLOV and grown in aerobic conditions to OD_{600} 0.6 in LB media. Later cells are transferred into minimal-M9 media and moved into anerobic conditions, induced with varying concentrations of Fcn(O) and PYO and phiLOV fluorescence is measured via flow cytometer over 6 hours. As controls, pSC-S108gRNA plasmid expressing just the 108 gRNA is used for *phiLOV* activation. **(C)** qPCR data indicating repression of *soxS* by S1-108 gRNA from the pSCSoxS-S1-108gRNA. Controls including just the 108 gRNA and the Control-108gRNA hybrid do not repress *soxS* upon PYO induction. **(D)** pSCSoxS-S1-108gRNA is transformed along with dCas9-ω and pTT01 reporter plasmid into NB101 cells. At OD_{600} 0.6, cells in minimal-M9 media are induced with 5mM Fcn(O) and 5 μM PYO under anaerobic conditions and phiLOV fluorescence is measured via a flow cytometer over 6 hours. As controls, pS1gRNA plasmid expressing just the S1 gRNA is used as a control. For **(B)** and **(C)**, n=3 independent experimental replicates and for **(D)**, n=2 independent experimental replicates. Replicates are represented by circles and height of the bars indicate mean. For **(B)** and **(C)**, error bars indicate standard deviation. Source data is provided separately.

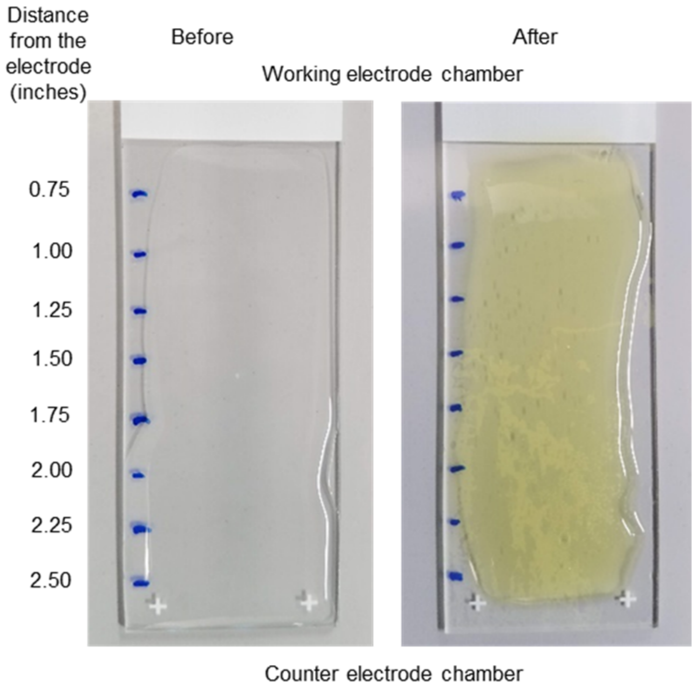

Supplementary Figure 13: Image of the gelatin hydrogels exposed to the electrochemical gradients

Gelatin hydrogel encapsulating W3110 populations containing pSoxS-phiLOV plasmids and coated onto glass slides. Glass slides are placed in the electrophoresis device containing minimal-M9 media supplemented with Fcn(R) and PYO and an oxidation potential of +0.3 V is applied across the device for 8 hours. Before and after images of hydrogels indicate phiLOV fluorescence after exposure to Fcn(O) gradients for 8 hours. This is a representative image only. In Fig. 5C and 5D, three independent experiments were performed.

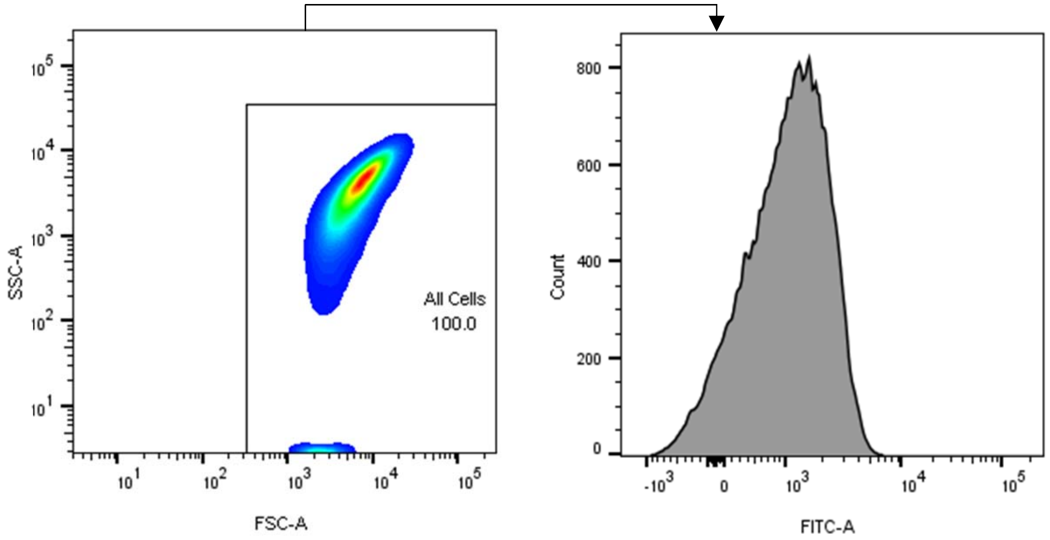

Supplementary Figure 14: Image of the flow cytometry gating scheme

Gating scheme indicates the FSC and SSC settings used to capture the cells. All cells totaling 50,000 were captured and used for PhiLOV fluorescence measurements. The average fluorescence is measured and used for analysis.

Supplementary Methods

Supplementary Table 1: Table of strains and plasmids

Strain	Relevant Genotype and Property	Reference
NB101	ZK126[7] Δ*rpoZ*	This study
JEN202	MG1655, Δ*rpoZ*	[2]
DJ901	DlacU169 rpsL DsoxRS901(GC4468 Δ*soxRS*)	[8]
**Plasmids**	**Relevant Genotype and Property**	**Reference**
pWJ89	pZS*24-MCS1, PAM-rich 5' UTR region, J23117, *gfp-mut2*	[2]
pWJ66	pACYC184(Cm^r) with tracrRNA, *cas9*(D10A, H840A)-ω, repeat-BsaI spacer-repeat	[2]
p108gRNA	108-spacer (from Bikard *et.al.*) in pgRNA-bacteria (Addgene plasmid # 44251) with pBR322 origin, Amp^r, J23119 promoter	This study
pControlgRNA	Control spacer (from Bikard *et.al.*) in pgRNA-bacteria (Addgene plasmid # 44251) with pBR322 origin, Amp^r, J23119 promoter	This study
pdCas9-ω	ω was inserted into C termini of dCas9 in pdCas9-bacteria (Addgene plasmid # 44249), p15A, pLtetO-1, Cm^r	This study
pdCas9-2ω	Two ω's was inserted into C termini of dCas9 in pdCas9-bacteria (Addgene plasmid # 44249), p15A, pLtetO-1, Cm^r	This study
pdCas9-3ω	Three ω's was inserted into C termini of dCas9 in pdCas9-bacteria (Addgene plasmid # 44249), p15A, pLtetO-1, Cm^r	This study
pdCas9-ω_{ssrA}	ssrA protein degradation tag added to the C termini of ω in pdCas9-ω	This study
pIntdCas9-ω	pdCas9-ω with engineered Tet promoter and dCas9 RBS sites	This study
pIntdCas9-ω_{ssrA}	ssrA protein degradation tag added to the C termini of ω in pIntdCas9-ω	This study
pNC-gRNA-1, 4 &5	p108gRNA with single point mutations in the seed region of 108 spacer	This study
pTrc-108gRNA	108 gRNA sequence inserted in pTrcHisA plasmid (Invitrogen)	This study
pSoxS-108gRNA	108 gRNA sequence inserted into SoxS promoter in pTT01[9]	This study
pSC-S108gRNA	pSoxS-108gRNA, pSC101*, Kan^r	This study
pMC-GFP	pWJ89 with pBR322 and Amp^r instead of pSC101* and Kan^r	This study
pMC-LasI	*lasI* instead of *gfpmut2* in pMC-GFP	This study
pMC-phiLOV	*phiLOV* instead of gfpmut2 in pMC-GFP	This study
pS1gRNA, pS2gRNA and pS3 gRNA	SoxS specific gRNA spacers (S, S2 and S3) inserted into pgRNA-bacteria (Addgene plasmid # 44251) with pBR322 origin, Amp^r, BBa_J23119 promoter	This study
pSoxS-phiLOV	*phiLOV* under SoxS promoter, pBR322, Amp^r	[9]
pSoxS-phiLOV:pS1gRNA	*E. coli* SoxS specific S1 gRNA under J23119 promoter in pSoxS-phiLOV	This study
pSCSoxS-S1gRNA	S1 gRNA under SoxS promoter, pSC101*, Kan^r	This study

pSCSoxS-S1-108gRNA	HH-S1gRNA-HDV-HH-108gRNA-HDV under SoxS promoter, pSC101*, Kan^r	This study
pSCSoxS-Control-108gRNA	HH-ControlgRNA-HDV-HH-108gRNA-HDV under SoxS promoter, pSC101*, Kan^r	This study
pSCSoxS-108-S1gRNA	HH-108gRNA-HDV-HH-S1gRNA-HDV under SoxS promoter, pSC101*, Kan^r	This study
pSoxS-phiLOV:pS3gRNA	*Salmonella enterica soxS* specific S3 gRNA under J23119 promoter in pSoxS-phiLOV	This study

Supplementary Table 2: Table of Primers

Primer Name	Sequence (5'-3')	Features
**p108gRNA**		
N1	tccggcctgcagccagttttagagctagaaatagcaag	Amplifies 108 spacer with pgRNA-bacteria [for p108gRNA]
N2	tcttccacaacacgcactagtattatacctaggactg	
**pControlgRNA**		
N3	aagctcaaaggtctcgttttagagctagaaatagcaag	Amplifies Control spacer with pgRNA-bacteria [for pControlgRNA]
N4	ccgagactggtctcaactagtattatacctaggactg	
**pTrc- ω, pTrc- 2ω and pTrc- 3ω**		
N5	gctagcctcgagggtggtggttcagcacgcgtaactgttcaggac	Nhe-1, Xho-1; amplifies ω subunit-1
N6	tgcagatcttgaaccaccaccacgacgaccttcagcaatagc	Bgl-2; amplifies ω subunit-1
N7	tcaagatctgcacgcgtaactgttcaggac	Bgl-2; amplifies ω subunit-2
N8	gcgaattcagaaccaccaccacgacgaccttcagcaatagcgg	GGGS linker, EcoR-1; amplifies ω subunit-2
N9	gaattcgcacgcgtaactgttcaggac	EcoR-1; amplifies ω subunit-3
N10	aagcttttaacgacgaccttcagcaatagcg	Hind-3; amplifies ω subunit-3
**pdCas9- ω, 2ω, 3ω**		
N11	gaaggtcgtcgttaaggatctccaggcatcaaataaaacgaaagg	Stop site; amplifies vector pdCas9 [for pdCas9- ω, 2ω, 3ω]
N12	aacagttacgcgtgctgaaccaccaccgtcacctcctagctgactcaaa tcaatgc	GGGS linker, overlaps ω, amplifies vector pdCas9[for pdCas9- ω, 2ω, 3ω]
**pdCas9-ω_{ssrA}**		
N13	gctaggaggtgacggtggtggttcagca	Amplifies ω subunit-3 [for pdCas9-ω_{ssrA}]
N14	atgcctggagatccttaagcagccagagcgtagttttcgtcgttagcagc acgacgaccttcagc	Amplifies ω subunit-3 with ssrA tag and stop codon [for pdCas9-ω_{ssrA}]
**pdIntCas9- ω and pdIntCas9- ω_{ssrA}**		
N15	atcggcacaaatagcgtcgg	Amplifies pdCas9 excluding pLtetO-1 [for pIntdCas9-ω and pIntdCas9-ω_{ssrA}]
N16	cattagagctgcttaatgaggtcgg	
**pTrc-108gRNA**		
N17	aattaaagaggtatatattaagcgtgttgtggaagatcc	Amplifies 108 gRNA, overlaps pTrcHisA [for pTrc-108gRNA]
N18	tctcatccgccaaaacagccaaaaaagcaccgactcgg	
N19	ggatcttccacaacacgcttaatatatacctctttaatt	Amplifies pTrcHisA, overlaps 108 gRNA spacer-scaffold [for pTrc-108gRNA]
N20	ccgagtcggtgcttttttggctgttttggcggatgaga	
**pSoxS-108gRNA**		
N21	catgtttgacagcttatcatcgatattaaaaaagcaccgactcggt	Amplifies 108 gRNA, overlaps pSoxS-phiLOV [for pSoxS-108gRNA]
N22	tgaaaagaggcagatttgcgtgttgtggaagatccg	
N23	cggatcttccacaacacgcaaatctgcctcttttca	Amplifies pSoxS-phiLOV, overlaps 108 gRNA spacer-scaffold [for pSoxS-108gRNA]
N24	accgagtcggtgcttttttaatatcgatgataagctgtcaaacatg	
**pSC-S108gRNA**		
N25	ctacgctctggctgcttaaggatcccatggtacgcgtgctagagg	Amplifies pSC101*, Kan^r backbone from pWJ89 [for pSC-S108gRNA]
N26	ttttcgtcgttagcagctttgtatagttcatccatgccatgtgtaatcccag	
N27	aaatctgcctcttttcagtg	Amplifies SoxR-pSoxS-108gRNA [for pSC-S108gRNA]
N28	atcgatgataagctgtcaaa	

**pMC-GFP**		
N29	tacaagagccataagaacctctacaaactctttttgtttatttttctaaataca ttcaaatatgtatc	Amplifies Amp-pBR322 cassette [for pMC-GFP]
N30	caggatgaggatcgtttcgccgcgttgctggcgtttttc	
N31	aggttcttatggctcttg	Amplifies pWJ89 excluding pSC101*, Kan^r [for pMC-GFP]
N32	gcgaaacgatcctcatcc	
**pMC-LasI**		
N33	ggatcccatggtacgcgtg	Amplifies pMC-GFP excluding *gfpmut2* [for pMC-LasI]
N34	ctagatttctcctctttaaaggaattcgc	
N35	tttaaagaggagaaatctagatgatcgtacaaattggtcgg	Amplifies *lasI* [for pMC-LasI]
N36	gcacgcgtaccatgggatcctcatgaaaccgccagtcg	
**pMC-phiLOV**		
N37	tttaaagaggagaaatctagatgattgaaaaaagctttgtgattac	Amplifies *phiLOV* [for pMC-phiLOV]
N38	gcacgcgtaccatgggatccttacacatgatcgctgcc	
N39	ctagatttctcctctttaaaggaattcgc	Amplifies pMC-GFP excluding *gfpmut2* [for pMC-phiLOV]
N40	ggatcccatggtacgcgtg	
**pS1gRNA and pS2gRNA**		
N41	gaataattttctgatgttttagagctagaaatagc	Replaces 108 gRNA with S1gRNA [for pS1]
N42	aggatcttatcgcatactagtattatacctaggac	
N43	tgaataattttctgagttttagagctagaaatagc	Replaces 108 gRNA with S2 gRNA [for pS2]
N44	ggatcttatcgcatgactagtattatacctaggac	
**pSoxS-phiLOV:pS1gRNA**		
N45	ggagtcgcataagggagagcgaattctaaagatctttgacagctagc	Amplifies S1 gRNA with J23119 promoter
N46	gaaggctctcaagggcatcgggcccagtctttcgactg	
N47	cgatgcccttgagagccttc	Amplifies pSoxS-phiLOV backbone
N48	cgctctcccttatgcgac	
**pSCSoxS-S1gRNA**		
N49	gaataattttctgatgttttagagctagaaatagc	Replaces 108 spacer with S1 spacer in pSC-S108gRNA [for pSCSoxS-S1gRNA]
N50	aggatcttatcgcataagcttaaatctgcctcttttc	
**pSoxS-phiLOV:pS3gRNA**		
N51	caatccattcgataagttttagagctagaaatagcaag	Replaces 108 spacer with S3 spacer in pSC-S108gRNA [for pSoxS-phiLOV:pS3gRNA]
N52	atgaacatatcgaccacctaggactgagctagc	

List of sequences and spacers

Spacer Name: **Spacer sequence (5'-3')**

Spacer Control: tgagaccagtctcggaagctcaaaggtctc

Spacer 108: gcgtgttgtggaagatccggcctgcagcca

Spacer NC-1: gcgtgttgtggaagatccggcctgcagccc

Spacer NC-4: gcgtgttgtggaagatccggcctgcagcta

Spacer NC-5: gcgtgttgtggaagatccggcctgcagtca

Spacer S1: atgcgataagatcctgaataattttctgat

Spacer S2: catgcgataagatcctgaataattttctga

Spacer S3: ggtcgatatgttcatcaatccattcgataa

Synthetic Intergenic Region for pIntdCas9-ω and pIntdCas9-ω_{ssrA} (5'-3'):

Aagcagaggagcaaaagctcatttctgaagaggacttgttgcggaaacgacgagaacagttgaaacacaaacttgaacagctacgg aactcttgtgcgtaaggaaaagtaaggaaaacgattccttctaacagaaatgtcctgagcaatcacctatgaactgtcgactcgagcctc tatggattatcaccttggctgcaggccggatcttccacaacacgcacggtgttacattaggcataccggtcttgacagctagctcagtcct agggattgtgctagcgaattcctttaaagaggagaaatctagatg

HDV-S1gRNA-HH (5’-3’):

Atagcgggctcacccagaaacgctccatggtcccattcgccatgccgaagcatgttgcccagccggcgccagcgaggaggctggg
acctgccggccaaaagcaccgactcggtgccactttttcaagttgataacggactagccttattttaacttgctatttctagctctaaaacat
cagaaattattcaggatcttatcgcatgacgagcttactcgtttcgtcctcacggactcatcagatgcgaaagcttaaatctgcct

HDV-108gRNA-HH (5’-3’):

Gacagcttatcatcgatattccatggtcccattcgccatgccgaagcatgttgcccagccggcgccagcgaggaggctgggaccatg
ccggccaaaagcaccgactcggtgccactttttcaagttgataacggactagccttattttaacttgctatttctagctctaaaactggctgc
aggccggatcttccacaacacgcgacgagcttactcgtttcgtcctcacggactcatcaggcgtgtatagcgggctcacccagaaacg
ctccatg

HDV-ControlgRNA-HH (5’-3’):

Atagcgggctcacccagaaacgctccatggtcccattcgccatgccgaagcatgttgcccagccggcgccagcgaggaggctggg
accatgccggccaaaagcaccgactcggtgccactttttcaagttgataacggactagccttattttaacttgctatttctagctctaaaac
gagacctttgagcttccgagactggtctcagacgagcttactcgtttcgtcctcacggactcatcagtgagacaagcttaaatctgcct

Plasmids created using site-directed mutagenesis

p108gRNA and pControlgRNA: We prepared p108gRNA and pControlgRNA by swapping spacers in the pgRNA vector (Addgene plasmid # 44251) through site-directed mutagenesis and standard protocols (New England Bio labs).

pNC-gRNA 1, 4 and 5: We prepared non-cognate gRNA1, 4 and 5 (pNC-gRNA 1, 4 and 5) by site-directed mutagenesis as well by swapping spacers with p108gRNA.

pS1gRNA, pS2gRNA and pS3gRNA: We prepared plasmid pS1gRNA, pS2gRNA and pS3gRNA for *soxS* repression by swapping the 108-spacer sequence with spacers S1, S2 and S3 in p108gRNA.

pSCSoxS-S1gRNA: We prepared pSCSoxS-S1gRNA by swapping the 108spacer in pSC-S108gRNA with the S1gRNA.

Preparation of pdCas9-ω, pdCas9-2ω, and pdCas9-3ω

To create pdCas9-ω, pdCas9-2ω and pdCas9-3ω, we used a two-stage process. First, we PCR amplified ω1, ω2 and ω3 subunits separately and used pTrcHisA (Invitrogen) as a shuttle vector to add all the ω subunits. We added ω3 subunit into pTrcHisA through NEB

restriction enzymes EcoRI and Hind-III to create pTrc-ω. Then, we added the ω2 subunit to pTrc-ω through restriction digestion with enzymes Bgl-II and EcoRI to create pTrc-2ω. Then, we employed another round of restriction digestion with enzymes NheI and Bgl-II to add ω1 subunit to pTrc-2ω to create pTrc-3ω. Then independent gene cassettes, 1ω, 2ω and 3ω, were removed from the pTrc-1ω, pTrc-2ω and pTrc-3ω plasmids through restriction digestion with Xho-1 and EcoRI and Gibson assembled into the pdCas9-Bacteria vector (Addgene plasmid # 44249), amplified with primers N11 and N12 to create pdCas9-ω, pdCas9-2ω, and pdCas9-3ω, respectively. All ω subunits and dCas9 had a GGGS linker in between.

Preparation of plasmids with Gibson assembly

pdCas9-ω_{ssrA}: We PCR amplified the ω subunit in pTrc-ω using primers encoding the ssrA tag and Gibson-assembled into an amplicon derived from pdCas9-Bacteria and primers N11 and N12. pIntdCas9-ω and pIntdCas9-ω_{ssrA}: The sequence of the synthetic intergenic region is noted above in our list of sequences. We amplified plasmids pdCas9-ω and pdCas9-ω_{ssrA} with primers N 15 and N16 and Gibson assembled these into the synthetic intergenic region (gene fragment from IDT) to create pIntdCas9-ω and pIntdCas9-ω_{ssrA}.

pTrc-108gRNA: We PCR amplified the 108 gRNA sequence from p108gRNA plasmid and Gibson assembled into pTrcHisA (Invitrogen).

pSoxS-108gRNA: We PCR amplified 108 gRNA sequence from p108gRNA plasmid and Gibson assembled into pSoxS-phiLOV[9].

pSC-S108gRNA: We replaced the Amp^r cassette and pBR322 origin in pSoxS-108gRNA with a Kan^r and pSC101* cassette from pWJ89[2] though PCR and Gibson assembly.

pMC-GFP: We replaced the Kan^r and pSC101* cassette in pWJ89 with an Amp^r and pBR322 cassette from pgRNA though PCR and Gibson assembly.

pMC-phiLOV: We replaced *gfpmut2* in pMC-GFP with *phiLOV* from pSoxS-phiLOV though PCR and Gibson assembly.

pMC-LasI: We replaced *gfpmut2* in pMC-GFP with *lasI* from pET200-*lasI* (lab stocks).

pSoxS-phiLOV:pS1gRNA and pSoxS-phiLOV:pS3gRNA: We PCR amplified the S1 gRNA and J23119 promoter from pS1 gRNA and Gibson assembled into pSoxS-phiLOV. Later, we replaced the S1 gRNA from pSoxS-phiLOV:pS1gRNA with S3 gRNA to create pSoxS-phiLOV:pS3gRNA.

pSCSoxS-S1-108gRNA and pSCSoxS-Control-108gRNA: First, we synthesized gene fragments encoding HH-gRNA-HDV for 108 gRNA, Control gRNA and S1 gRNA. Additionally, we prepared a PCR amplicon excluding the 108 gRNA from pSC-S108gRNA and used it as vector to insert HH-gRNA-HDV sequences under the SoxS promoter. pSCSoxS-S1-108gRNA contained HH-S1gRNA-HDV and HH-108gRNA-HDV, in that order from the SoxS promoter; pSCSoxS-Control-108gRNA contained HH-ControlgRNA-HDV and HH-108gRNA-HDV, in that order from the SoxS promoter and pSCSoxS-108-S1gRNA contained HH-108gRNA-HDV and HH-S1gRNA-HDV, in that order from the SoxS promoter.

Supplementary references:

1 Cress, B. F. *et al.* Rapid generation of CRISPR/dCas9-regulated, orthogonally repressible hybrid T7-lac promoters for modular, tuneable control of metabolic pathway fluxes in Escherichia coli. *Nucleic Acids Research* **44**, 4472-4485, doi:10.1093/nar/gkw231 (2016).

2 Bikard, D. *et al.* Programmable repression and activation of bacterial gene expression using an engineered CRISPR-Cas system. *Nucleic Acids Research* **41**, 7429-7437, doi:10.1093/nar/gkt520 (2013).

3 Reisch, C. R. & Prather, K. L. J. The no-SCAR (Scarless Cas9 Assisted Recombineering) system for genome editing in Escherichia coli. **5**, 15096, doi:10.1038/srep15096

https://www.nature.com/articles/srep15096#supplementary-information (2015).

4 Scott, W. G., Murray, J. B., Arnold, J. R. P., Stoddard, B. L. & Klug, A. Capturing the Structure of a Catalytic RNA Intermediate: The Hammerhead Ribozyme. *Science* **274**, 2065-2069, doi:10.1126/science.274.5295.2065 (1996).

5 Nakano, S.-i., Chadalavada, D. M. & Bevilacqua, P. C. General Acid-Base Catalysis in the Mechanism of a Hepatitis Delta Virus Ribozyme. *Science* **287**, 1493-1497, doi:10.1126/science.287.5457.1493 (2000).

6 Tang, X. *et al. A CRISPR–Cpf1 system for efficient genome editing and transcriptional repression in plants*. Vol. 3 (2017).

7 Connell, N., Han, Z., Moreno, F. & Kolter, R. An E. coli promoter induced by the cessation of growth. *Molecular Microbiology* **1**, 195-201, doi:10.1111/j.1365-2958.1987.tb00512.x (1987).

8 Greenberg, J. T., Monach, P., Chou, J. H., Josephy, P. D. & Demple, B. Positive Control of a Global Antioxidant Defense Regulon Activated by Superoxide-Generating Agents in Escherichia coli. *Proceedings of the National Academy of Sciences of the United States of America* **87**, 6181-6185 (1990).

9 Tschirhart, T. *et al.* Electronic control of gene expression and cell behaviour in Escherichia coli through redox signalling. **8**, 14030, doi:10.1038/ncomms14030

https://www.nature.com/articles/ncomms14030#supplementary-information (2017).
