# Supplementary material for: A redox-based electrogenetic CRISPR system to connect with and control biological information networks
Source: Nat Commun. 2020 May 15;11:2427. doi: 10.1038/s41467-020-16249-x (PMC7228920; doi:10.1038/s41467-020-16249-x)
Supplement: Supplementary file 2 — Reporting Summary [file 41467_2020_16249_MOESM2_ESM.pdf]

nature research

Corresponding author(s): Dr. William E. Bentley

Last updated by author(s): 04/05/2020

Reporting Summary

Nature Research wishes to improve the reproducibility of the work that we publish. This form provides structure for consistency and transparency in reporting. For further information on Nature Research policies, seeAuthors & Referees and theEditorial Policy Checklist .

Statistics

For all statistical analyses, confirm that the following items are present in the figure legend, table legend, main text, or Methods section.

n/a	Confirmed	
☐	☒	The exact sample size (*n*) for each experimental group/condition, given as a discrete number and unit of measurement
☐	☒	A statement on whether measurements were taken from distinct samples or whether the same sample was measured repeatedly
☒	☐	The statistical test(s) used AND whether they are one- or two-sided<br>*Only common tests should be described solely by name; describe more complex techniques in the Methods section.*
☒	☐	A description of all covariates tested
☒	☐	A description of any assumptions or corrections, such as tests of normality and adjustment for multiple comparisons
☐	☒	A full description of the statistical parameters including central tendency (e.g. means) or other basic estimates (e.g. regression coefficient) AND variation (e.g. standard deviation) or associated estimates of uncertainty (e.g. confidence intervals)
☒	☐	For null hypothesis testing, the test statistic (e.g. *F*, *t*, *r*) with confidence intervals, effect sizes, degrees of freedom and *P* value noted<br>*Give P values as exact values whenever suitable.*
☒	☐	For Bayesian analysis, information on the choice of priors and Markov chain Monte Carlo settings
☒	☐	For hierarchical and complex designs, identification of the appropriate level for tests and full reporting of outcomes
☒	☐	Estimates of effect sizes (e.g. Cohen's *d*, Pearson's *r*), indicating how they were calculated

Our web collection on statistics for biologists contains articles on many of the points above.

Software and code

Policy information about availability of computer code

Data collection	BD FacsDiva software was used to collect data from the flow cytometer. SoftMax Pro V 5.3 (Molecular Devices) was used to collect data from the plate reader. Laser scanning microscope was used for imaging of cells in Fig. 5c and 5d.
Data analysis	The raw data was analyzed using MS-Excel, data is exported to Graphpad Prism 8 for generation of all graphs. FlowJo 10.6.2 is used to create Supplemental Figure 13. ImageJ was used for quantification of fluorescence in Fig. 5c and 5d.

For manuscripts utilizing custom algorithms or software that are central to the research but not yet described in published literature, software must be made available to editors/reviewers. We strongly encourage code deposition in a community repository (e.g. GitHub). See the Nature Research guidelines for submitting code & software for further information.

Data

Policy information about availability of data

All manuscripts must include a data availability statement. This statement should provide the following information, where applicable:

- Accession codes, unique identifiers, or web links for publicly available datasets
- A list of figures that have associated raw data
- A description of any restrictions on data availability

The data that support the findings of this study are available from the corresponding author upon reasonable request.

Field-specific reporting

Please select the one below that is the best fit for your research. If you are not sure, read the appropriate sections before making your selection.

Life sciences study design

All studies must disclose on these points even when the disclosure is negative.

Sample size	At least 3 sets of experiments were run for biological experiments that demonstrate small molecule as well as electrical induction of SoxS promoters for gRNA expression, CRISPRa of fluorescent reporters and LasI, AI-1 activities from reporter cells, repression of genomic SoxS in both E. coli and Salmonella leading to enhanced fluorescent protein expression in both strains. For supplementary figures 8 and 10, the purpose of the experiments was to characterize relative trends across various concentrations - 2 sets of experiments were performed to observe the trends. Each has internal controls. For supplementary figure 9, its purpose was to re-confirm data shown in Figs. 3B and 3C, hence 2 biological replicates were deemed sufficient.
Data exclusions	No data was excluded from the analyses.
Replication	The figures in the main text and the supplemental section demonstrate the reproducibility of the system and its quantified results. Experiments were carried out using the same overnight cultures for intra-day experiments and results are given with standard errors and standard deviations representing the variations that are inherent to the biological system as well as the measurement techniques used to obtain the data.
Randomization	Randomization was not relevant to this study since the same overnight culture was used for all conditions in a given experiment.
Blinding	Blinding was not relevant to this study.

Reporting for specific materials, systems and methods

We require information from authors about some types of materials, experimental systems and methods used in many studies. Here, indicate whether each material, system or method listed is relevant to your study. If you are not sure if a list item applies to your research, read the appropriate section before selecting a response.

Materials & experimental systems

n/a	Involved in the study	
☒	☐	Antibodies
☒	☐	Eukaryotic cell lines
☒	☐	Palaeontology
☐	☒	Animals and other organisms
☒	☐	Human research participants
☒	☐	Clinical data

Methods

n/a	Involved in the study	
☒	☐	ChIP-seq
☐	☒	Flow cytometry
☒	☐	MRI-based neuroimaging

Animals and other organisms

Policy information about studies involving animals; ARRIVE guidelines recommended for reporting animal research

Laboratory animals	The study did not involve animals. E. coli W3110 and W3110 derivate strains were used for experiments. E. coli strain TOP10 was used for cloning. All strains are listed and described in detail in the supplemental section.
Wild animals	The study did not involve wild animals.
Field-collected samples	The study did not involve samples collected from the field.
Ethics oversight	No ethical approval or guidance in the design of this study by an organization was required. Only commonly used E. coli strains were used in this study. All experiments were carried out in accordance with University of Maryland regulations.

Note that full information on the approval of the study protocol must also be provided in the manuscript.

Flow Cytometry

Plots

Confirm that:

☐ The axis labels state the marker and fluorochrome used (e.g. CD4-FITC).

☐ The axis scales are clearly visible. Include numbers along axes only for bottom left plot of group (a 'group' is an analysis of identical markers).

☐ All plots are contour plots with outliers or pseudocolor plots.

☒ A numerical value for number of cells or percentage (with statistics) is provided.

Methodology

Sample preparation	We took bacterial cells at various time points, spun the cells down via centrifugation, re-suspended the cells in 2% paraformaldehyde in phosphate buffered saline, pH 7.4 and fixed the cells for a minimum of 15 mins and used for measurements.
Instrument	BD FACSCanto(San Jose, CA)
Software	BD FacsDiva software was used for data collection. MS-Excel was used for analysis
Cell population abundance	We used a constant cell number of 50,000 cells in all cases.
Gating strategy	We did not use any gating for fluorescence measurements. We simply used flow cytomoter to measure fluorescence of the whole population. We used a constant FSC and SSC setting for all experiments involving E. coli and Salmonella. A supplemental figure S13 is added in this regard.

☒ Tick this box to confirm that a figure exemplifying the gating strategy is provided in the Supplementary Information.
